# Supplementary figures and images for: Two Approaches to Triple Antithrombotic Therapy in Patients with Acute Coronary Syndrome and Non-Valvular Atrial Fibrillation Treated with Percutaneous Coronary Intervention: Which Is More Efficient and Safer?
Source: Diagnostics (Basel). 2023 Sep 26;13(19):3055. doi: 10.3390/diagnostics13193055 (PMC10572308; doi:10.3390/diagnostics13193055)

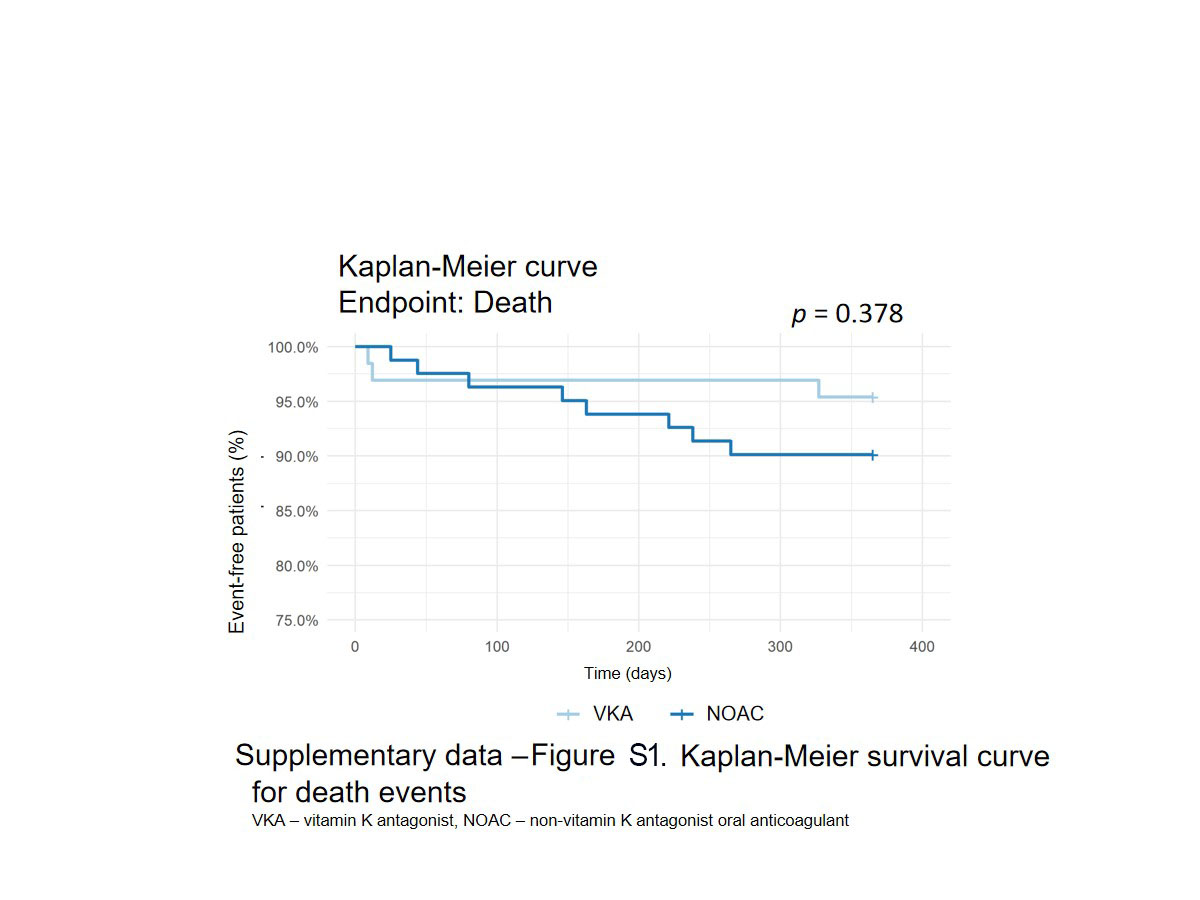

Supplement: Supplementary file 1 [file diagnostics-13-03055-s001.zip › Figure S1.jpg]

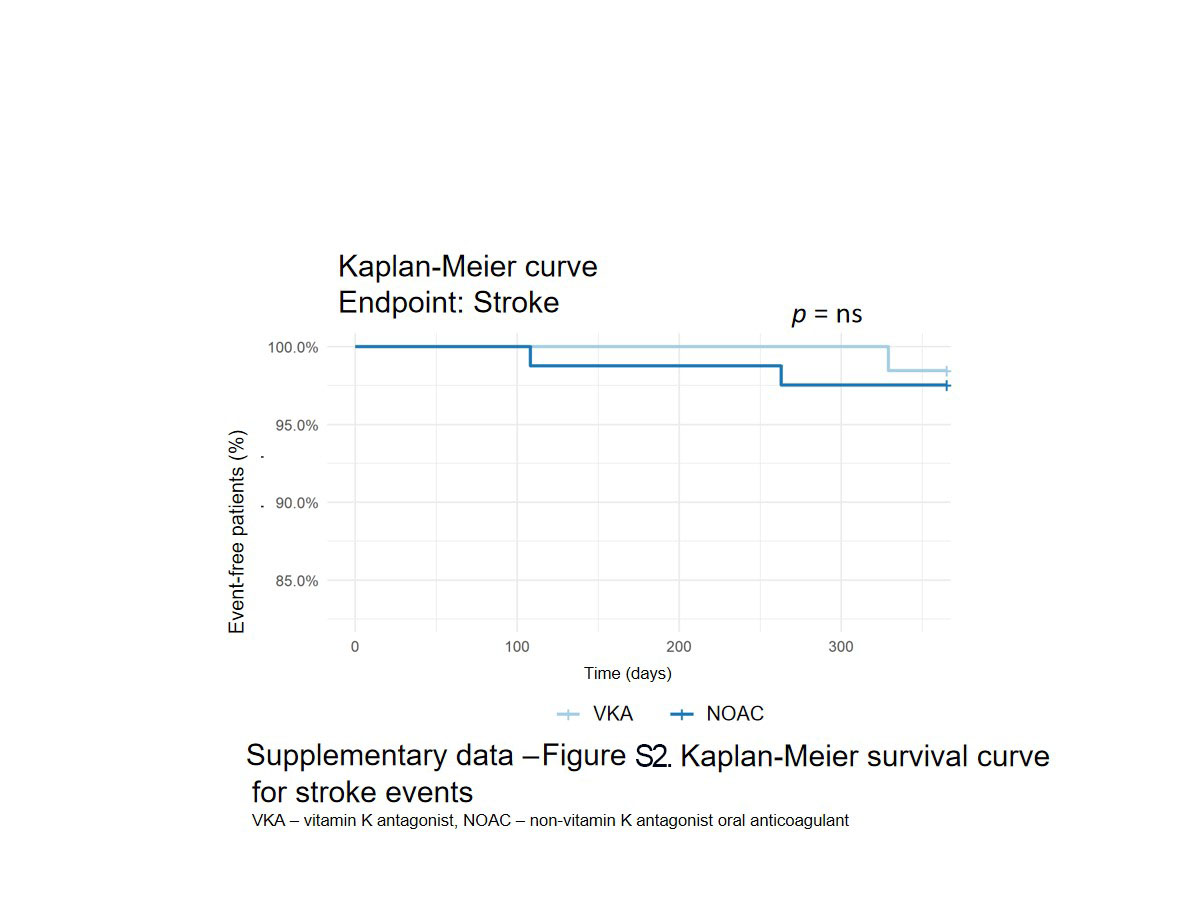

Supplement: Supplementary file 1 [file diagnostics-13-03055-s001.zip › Figure S2.jpg]

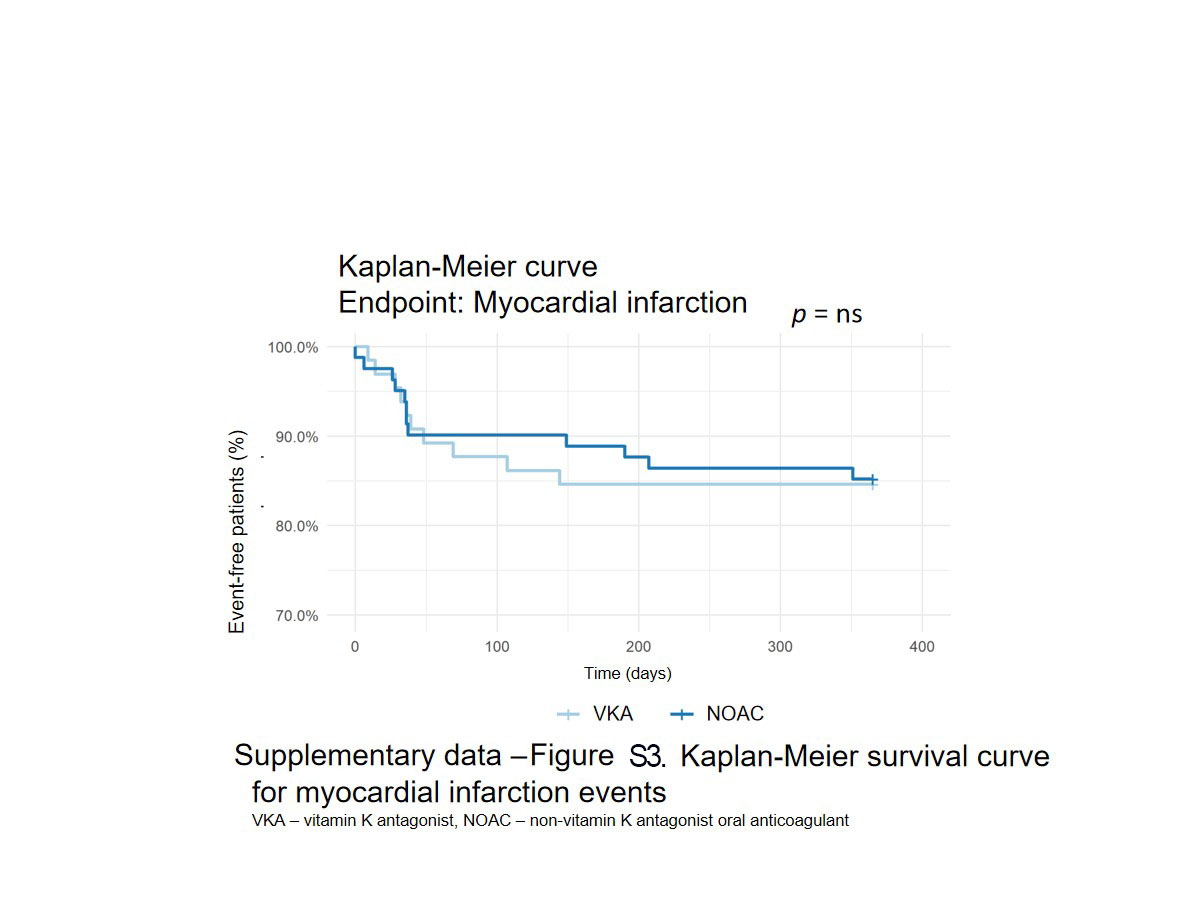

Supplement: Supplementary file 1 [file diagnostics-13-03055-s001.zip › Figure S3.jpg]

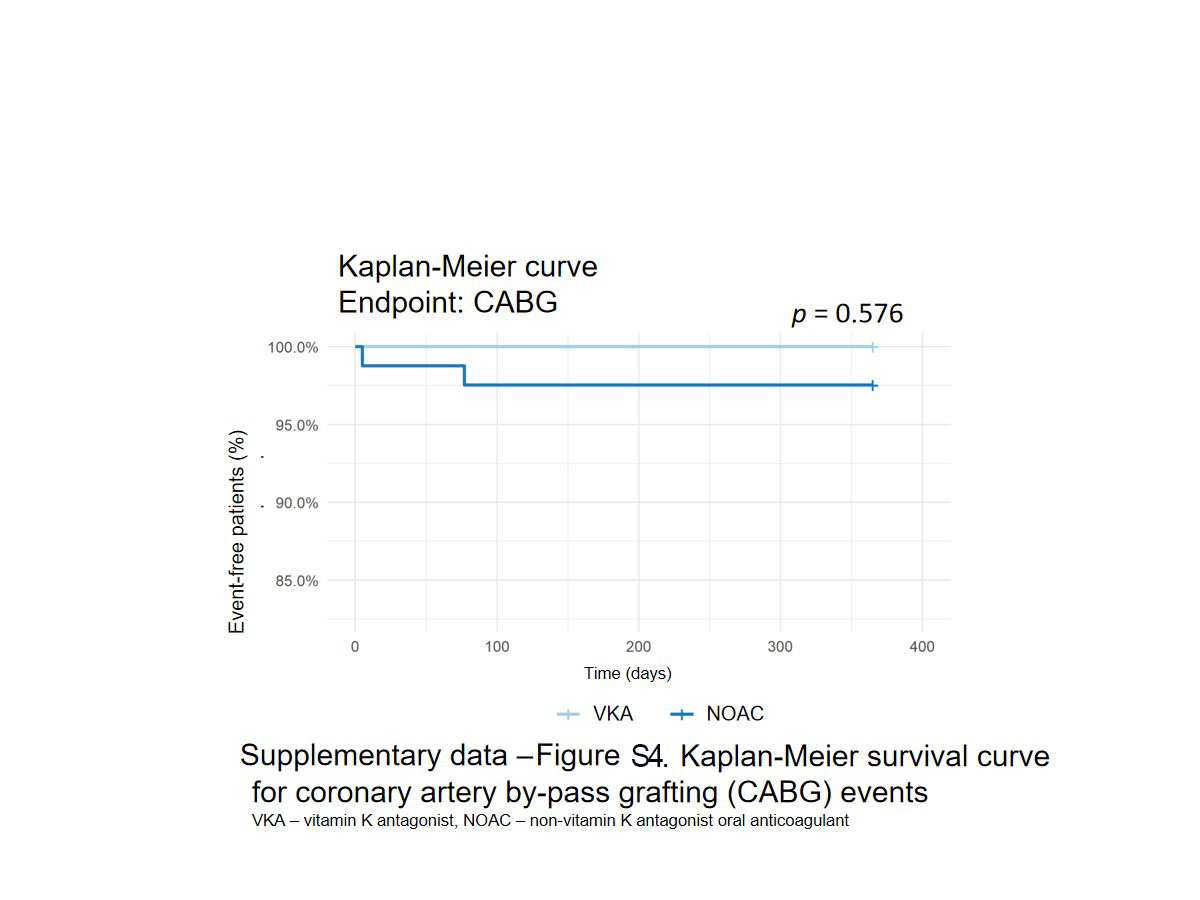

Supplement: Supplementary file 1 [file diagnostics-13-03055-s001.zip › Figure S4.jpg]

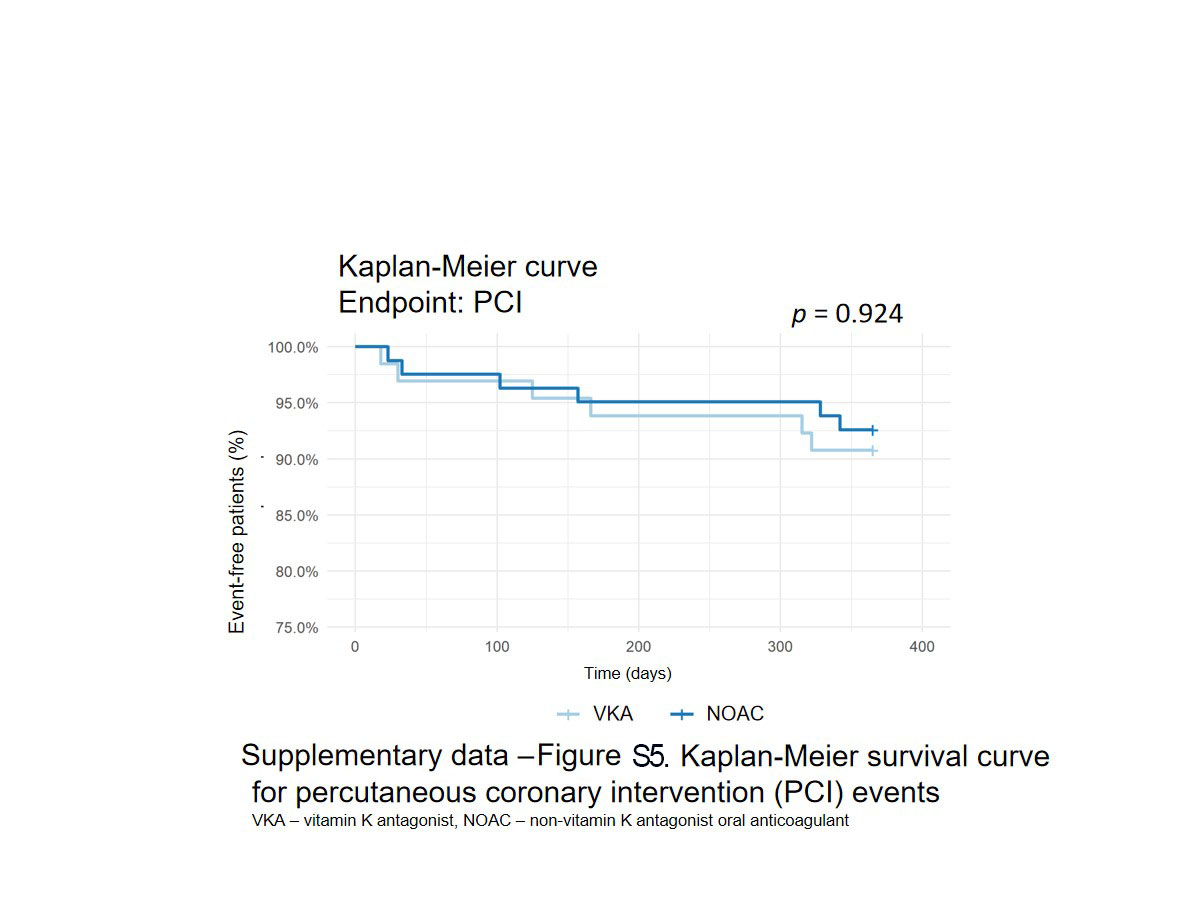

Supplement: Supplementary file 1 [file diagnostics-13-03055-s001.zip › Figure S5.jpg]

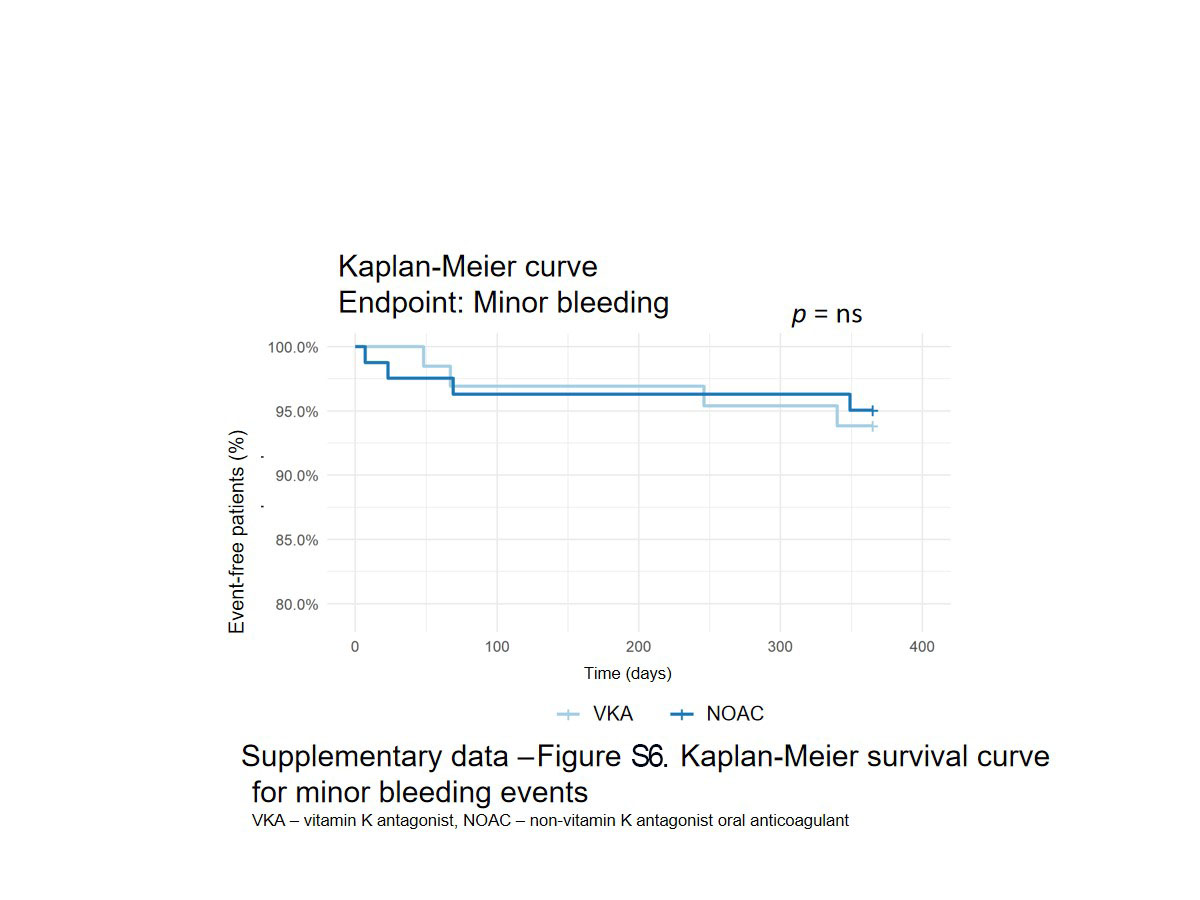

Supplement: Supplementary file 1 [file diagnostics-13-03055-s001.zip › Figure S6.jpg]

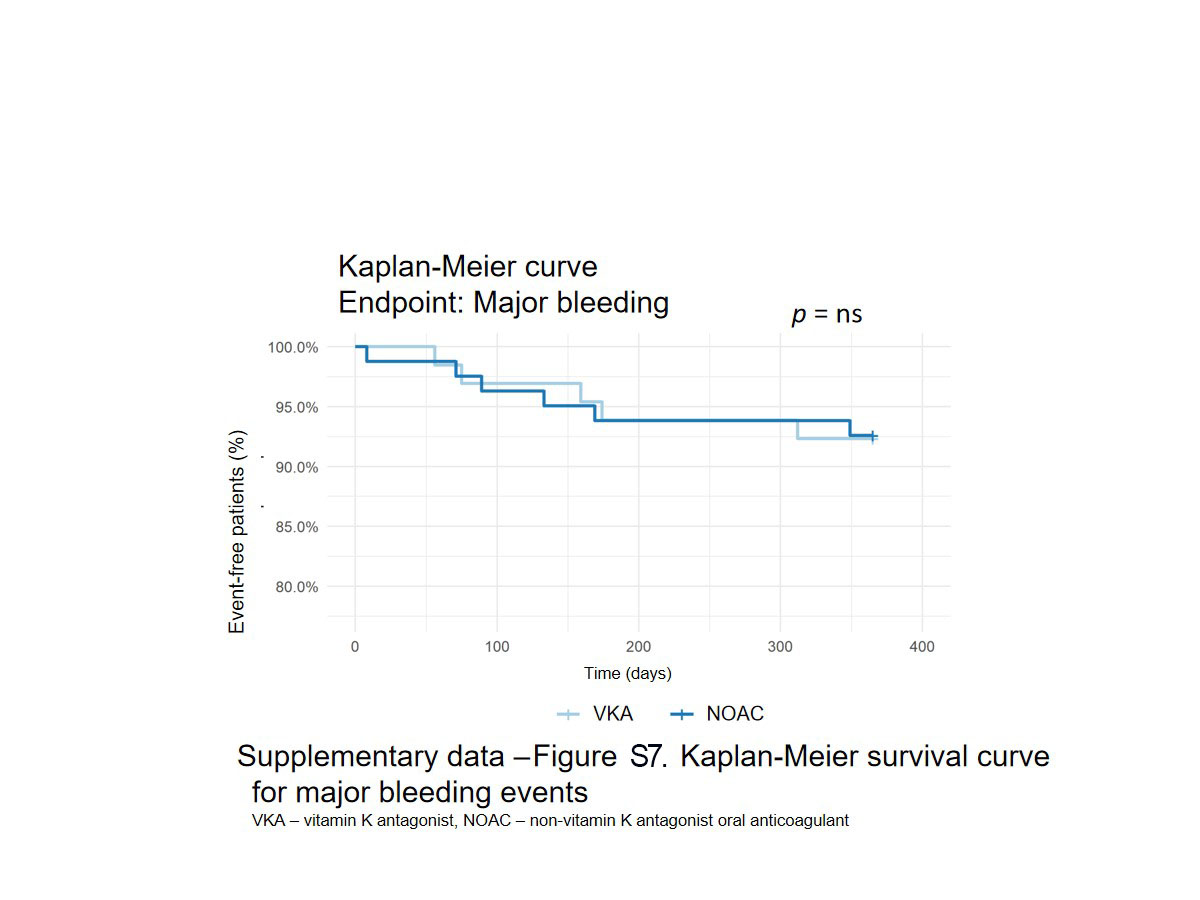

Supplement: Supplementary file 1 [file diagnostics-13-03055-s001.zip › Figure S7.jpg]

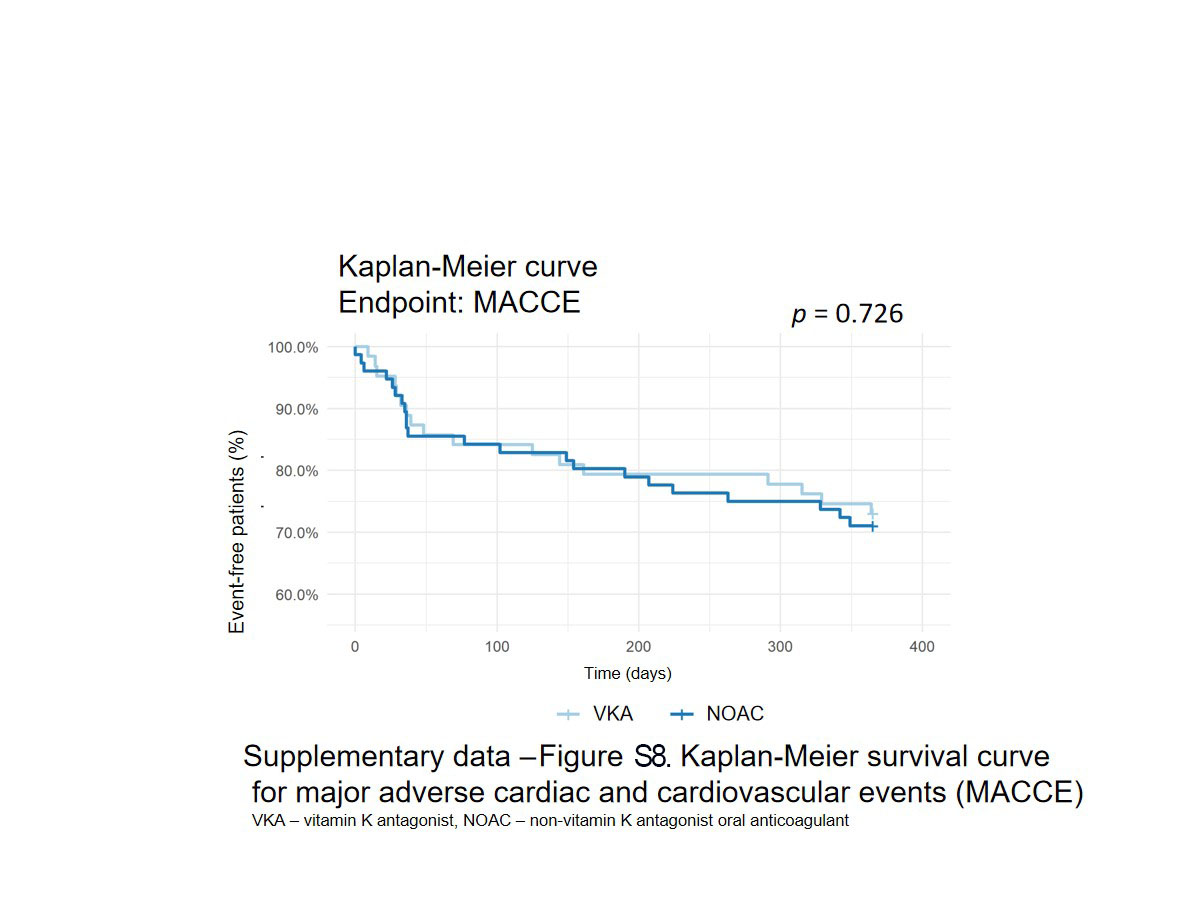

Supplement: Supplementary file 1 [file diagnostics-13-03055-s001.zip › Figure S8.jpg]

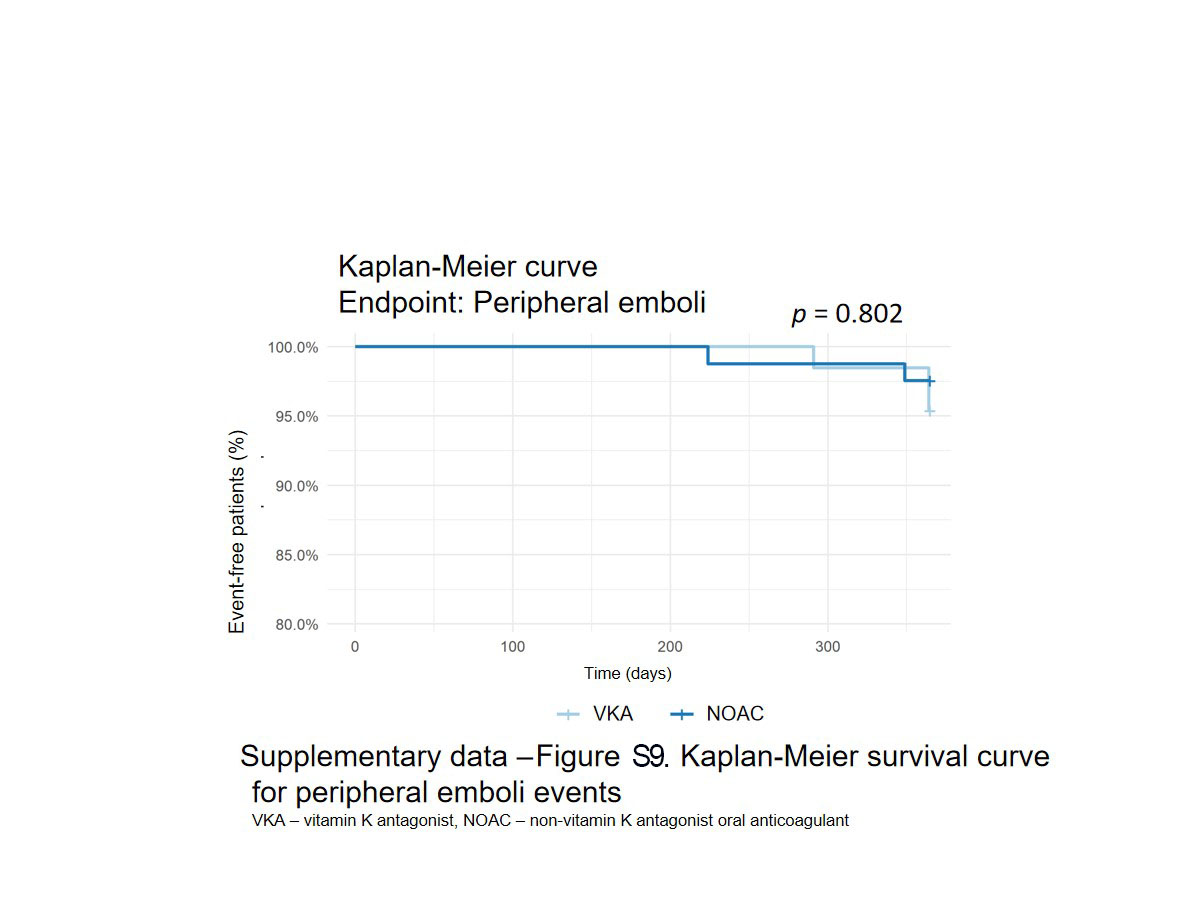

Supplement: Supplementary file 1 [file diagnostics-13-03055-s001.zip › Figure S9.jpg]
